# Spatial constraints on the diffusion of religious innovations: The case of early Christianity in the Roman Empire

**DOI:** 10.1371/journal.pone.0208744

**Published:** 2018-12-26

**Authors:** Jan Fousek, Vojtěch Kaše, Adam Mertel, Eva Výtvarová, Aleš Chalupa

**Affiliations:** 1 Institute of Computer Science, Masaryk University, Brno, Czech Republic; 2 Department for the Study of Religions, Faculty of Arts, Masaryk University, Brno, Czech Republic; 3 Faculty of Theology, University of Helsinki, Helsinki, Finland; 4 Department of Geography, Faculty of Science, Masaryk University, Brno, Czech Republic; 5 Faculty of Informatics, Masaryk University, Brno, Czech Republic; University at Buffalo - The State University of New York, UNITED STATES

## Abstract

Christianity emerged as a small and marginal movement in the first century Palestine and throughout the following three centuries it became highly visible in the whole Mediterranean. Little is known about the mechanisms of spreading innovative ideas in past societies. Here we investigate how well the spread of Christianity can be explained as a diffusive process constrained by physical travel in the Roman Empire. First, we combine a previously established model of the transportation network with city population estimates and evaluate to which extent the spatio-temporal pattern of the spread of Christianity can be explained by static factors. Second, we apply a network-theoretical approach to analyze the spreading process utilizing effective distance. We show that the spread of Christianity in the first two centuries closely follows a gravity-guided diffusion, and is substantially accelerated in the third century. Using the effective distance measure, we are able to suggest the probable path of the spread. Our work demonstrates how the spatio-temporal patterns we observe in the data can be explained using only spatial constraints and urbanization structure of the empire. Our findings also provide a methodological framework to be reused for studying other cultural spreading phenomena.

## Introduction

The spread of Christianity through the Roman Empire has been widely studied in the humanities relying especially on close reading of ancient literary sources [1, 2]. Because of the missing material evidence concerning Christianity until the third century [3], the spatial dimension of this spread has been studied only to a limited extent, primarily on the basis of spatial information found in early Christian texts [4, 5]. Although these sources are biased in details, we argue that they are reliable enough for the study of general patterns in the early spatial dissemination of Christianity.

Our work represents an extension of the pioneering work of Rodney Stark [6, 7]. In his 2006 book, Stark quantitatively explores and finds support for a few dozen hypotheses concerning the spread of Christianity, the cults of Cybele and Isis and the Jewish diaspora communities in the environment of the ancient Mediterranean and assumes that these processes share a number of common features. Here we are interested especially in two of his hypotheses: Hypothesis 3-2 (“The closer a city was to Jerusalem, the sooner a city had a Christian congregation”) and hypothesis 3-4 (“Larger cities had Christian congregations sooner than smaller cities”). When evaluating hypothesis 3-2, Stark focuses on 31 biggest cities of the Empire and their geographical distance from Jerusalem. Contrary to his approach, our analysis covers a substantially larger number of cities and we use a transportation model instead of geographical distance. Stark further evaluates the role of various cultural factors in the same process, like in hypothesis 3-3 (“Hellenic cities had Christian congregations sooner than did Roman cities”) and hypothesis 5-8 (“Cities with a significant Diasporan community were Christianized sooner than other cities”). Although we fully realize that cultural factors of this kind could be decisive for the spread of some other religious traditions in the ancient Mediterranean [8], we leave them out of our analysis as unnecessary for the explanation of the process of our interest.

By Christianization of a city we mean the emergence of the first Christian congregation in that city, counting ca. from a dozen to 200 adherents [9, 10]. We expect that later on other semi-autonomous congregations could have also emerged in the same city to experience an independent growth, but this is not our interest here. During the period of our interest, the total number of Christians was estimated to grow from 7,000–10,000 in 100 CE to 2,500,000–3,500,000 in 300 CE. [10]. According to this estimate, in the final stage under our scrutiny about 5 percent of the total population of the empire of 60 million inhabitants were Christianized, which corresponds to a growth rate of 34.2 percent per decade. While this is a rather conservative estimate [4, 6, 9–11], it still should be taken with reservation [12].

**Fig 1:**
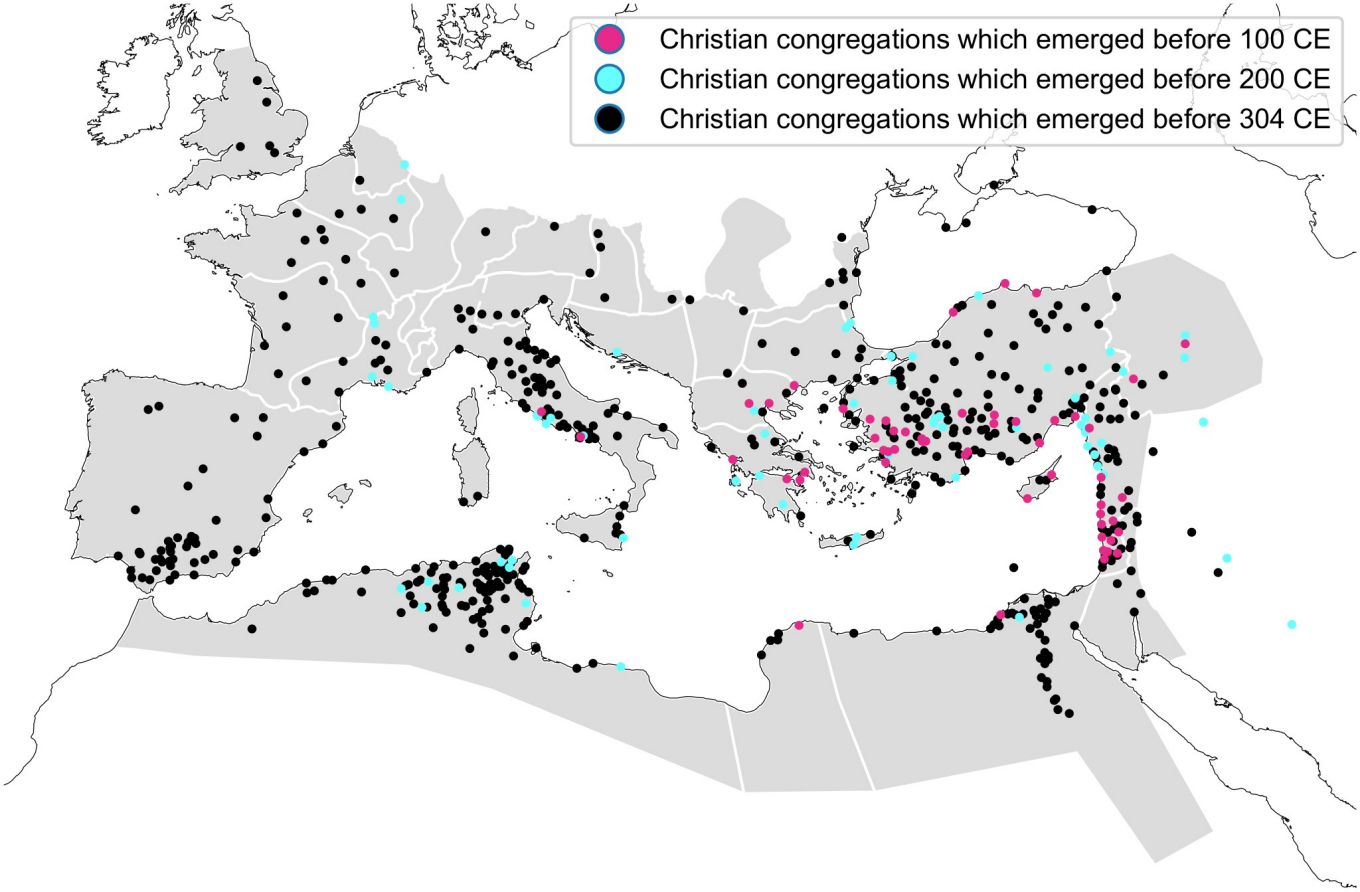
The spatio-temporal distribution of the documented presence of Christian congregations. The congregations from the three periods of interest [20] are shown on the outline of Roman provinces [21]. The map includes also congregations outside the empire.

Although it has been recently challenged that Christianity was mainly an urban phenomenon in its early existence and that we should not overlook the evidence for existence of numerous rural Christians [12], it remains probable that cities formed centers of Christian life and information exchange. Therefore, we start with data concerned with the presence of Christianity in cities (see Fig 1) and do not model the spatio-temporal pattern of the spread of Christianity as spatially continuous. Instead, we approach the spread of Christianity as a spatially nonuniform cascade in which the innovation can pass over intermediate small neighboring cities to larger cities which are more distant or, in our case, to which it is more expensive to travel.

To formalize this, we make use of the gravity model, which is widely used to model mobility flows and economic interactions [13–17]. A similar spreading pattern was previously studied with the help of the gravity model in the context of diffusion of linguistic innovations [18, 19].

We show that the spread of Christian congregations in the first three centuries follows to a large extent a gravity-guided diffusion model. We compare this model to corresponding static factors and to a simple spatial diffusion and demonstrate that our model is the best one in capturing the spatio-temporal pattern of the Christianization process. Compared to the empirical data, our model systematically overestimates the extent of Christianization in the area of modern Egypt and the neighborhood of the city of Carthage in the first two centuries, which can direct future qualitative research.

In recent years, network analysis has became increasingly popular both in archaeology and history [22], including the study of the spread of religions in the ancient Mediterranean [23]. However, most of this research has been rather heuristic or explanatory in nature and targeted primarily at humanities audiences. We take a step further by introducing a scientifically rigorous methodology designed to describe network diffusion processes driven by human mobility outside our own target domain. Since the presented method is easy to replicate and the model is abstract enough, they can also be adapted for studying other innovation diffusion processes in the past.

## Methods

### ORBIS transport model

In the structure of the transportation network in the ancient Mediterranean we rely on the data from the ORBIS project [24]. The ORBIS spatial network model consists of cities, roads, rivers and sea routes, roughly reflecting the conditions around 200 CE (see S1 Fig). It includes 648 cities and allows for calculations of distance, time duration and financial expense associated with different types of travel between these cities. For our purposes, we used the default cost from which the costs of particular means of transport (personal, cargo, etc.) are computed by multiplication by constant factor. Such coarse approximation of the nuances in calculation of the travel expenses is sufficient for our purposes, as the main effect of the model—substantial difference in cost per kilometer between land and maritime transport—is conserved in the default cost. The second parameter influencing the cost of travel in the ORBIS model is the season of travel. In our calculations, we used the minimal cost over the seasons for every route. The resulting transportation network is shown in the S2 Fig and the network data can be found in the S1 and S2 Files.

### Population estimates

As the precise data on population size of the cities in the ancient Mediterranean are unknown, they have to be derived by means of archaeological proxies. Here we draw on the estimates of the urbanization and city size offered by Wilson [25]. Wilson offers estimates for city sizes in two ways: there are 186 explicitly named cities and another 186 (sic!) not directly named cities given by number per given province. Although the Oxford Roman Economy Project lead currently by Wilson offers now a newer dataset on the size of Roman cities [26, 27], we use the one formerly published by Wilson, as it attempts to capture the proportional urbanization of the empire as a whole. Thus, when it is uncertain which settlement to include among the cities and which not, Wilson attempts to maintain the proportionality of the urbanization on a by-province basis by offering a number of cities with estimated average population size without specifying their name (e.g. by suggesting existence of 12 major cities with average population of 7000 in Britain).

From Wilson, we were able to map 121 explicitly named cities directly to the nodes of ORBIS. Then, the remaining explicit cities together with the cities not specified by title were assigned to the ORBIS nodes on a by-province basis to conserve the total population estimates for the given province. The population estimates for the cities in the province were assigned from largest to smallest according to the rank of the ORBIS node, which is derived from ORBIS source data on cities [28]. The list of all the ORBIS cities with the population estimate can be found in S3 File, and their position in the transportation network is shown in S2 Fig.

### Christian congregations

Despite the fact that there are some more up-to-date publications available [5, 29], we take the data on Christianization from the Atlas of the Early Christian World published in 1958 [20]. We chose this data as the maps in it explicitly attempt to pay balanced attention to all parts of the Roman world and because the maps also include contextual geographical information, which made their georeferencing substantially easier.

The data behind the maps are mainly based on information scattered over early Christian literary sources (the archaeological evidence is virtually non-existent before the third century [3]). Because of that, these kinds of maps have been widely criticized as tendentious and viewed with suspicion [30]. However, as we are interested more in overall spatial patterns than in regional details, this fact does not pose a serious problem for our work. Further, we assume that even when there are some newer data available, they do not challenge the general patterns as reflected in the work we rely on [31].

From the atlas, we coded three maps: “The Earliest Churches: Recorded Congregations of the First Century”, “The Church in the Second Century”, and “Churches Founded before the Persecution by Diocletian”. On this basis, we formed three point map layers representing Christian congregations claimed to emerge by the year 100 (N = 54), 200 (N = 111) and 304 (N = 594) respectively.

During the coding, wherever possible, we used georeferences from the Barrington Atlas of the Greek and Roman world [28]. The full list of the Christian congregations can be found in the S4 File. After that, we mapped the Christian congregations to the nearest ORBIS site to combine the spatially continuous evidence with the discrete nature of the transportation network. In the case where there were multiple congregations mapped to the same ORBIS site, we used the date of the earliest among those congregations. The result of this procedure was a matching of an ORBIS site with a year of the earliest documented Christian congregation located either directly at the site or in its immediate proximity. The entire mapping can be found in the S5 File.

### Gravity model

Gravity model has a long history of application to model mobility flows [13, 15] and economic interactions [14, 16]. We use it to estimate long-range social interactions, which can facilitate transfer of religious innovations. The estimated amount of interaction between two cities increases with their population sizes, and decreases with the distance between them:

$$\begin{array}{c} g_{nm}\propto \frac{N_{n}^{\alpha }N_{m}^{\beta }}{d^{\gamma }}, \end{array}$$ (1)

where *N*_*n*_ and *N*_*m*_ are the population sizes of the interacting cities and *d* is a measure of the distance. In all our analyses, the distance variable is substituted by the cost of travel based on the ORBIS model. The value of the parameters of the model varies across particular applications [32]. For the sake of simplicity, we assume symmetry of the interaction and attenuation of the effect of large nodes (*α* = *β* = 1/2). More variability is seen in the literature in the *γ* parameter, ranging from ≈ 0.5 to ≈ 3, possibly depending on the granularity of the modeled system [32]. Therefore, we opted for a mid-range *γ* = 2. However, we have found our results to be robust against variations in these exponents.

### Effective distance

The framework of effective distances enables us to model the dynamics and arrival times of a spreading process given just the network structure and flux of the carriers of the contagion in the network. It is based on the idea that the small fraction of traffic between two neighboring nodes is effectively equivalent to large distance and vice versa [33, 34].

Under this representation, the complex spatio-temporal geographical patterns exhibited by the diffusive processes on networks are turned into homogeneous traveling waves, which enables us to represent the dynamics of the spreading process without the need to realize the diffusive network model computationally. This compact representation however still allows one to reason about the origin, arrival times, and the spatio-temporal dynamics.

In our case, the flux *f*_*nm*_ is equal to the gravity *g*_*nm*_. The flux fraction $p_{nm}=\frac{f_{nm}}{\sum_{n}fnm}$ then gives for every neighbor *m* the fraction of the flux originating from node *n* and corresponds to the single-step probability of spreading the process from *n* to *m*. The flux fraction *p*_*nm*_ derived from the gravity model is a full network with numerous very weak links. To determine the significant links, we follow the procedure of Manitz et al. 35 by applying a threshold of $p_{nm}^{0}=1/M$ where *M* is the number of nodes in the network. The threshold corresponds to a flux fraction in a full network with randomly distributed traffic [36]. Resulting thresholded *p*_*nm*_ network had a sparsity of 25% (see S5 Fig).

The effective distance from *n* to a connected node *m* is then defined as *d*_*nm*_ = 1 − *logp*_*nm*_, and for path Γ = {*n*_1_, …, *n*_*k*_} the effective length is given by $\lambda \left(\Gamma \right)=\sum_{i=1}^{k-1}d_{n_{i},n_{i}+1}$. Using the effective path length, the effective distance between arbitrary nodes is defined as shortest path from *n* to *m*, i.e. *D*_*nm*_ = min_Γ_ λ(Γ). The shortest path tree Ψ_*n*_ then comprises the most probable paths of the spread of a process from the root node *n*.

## Results

### Static factors

**Fig 2:**
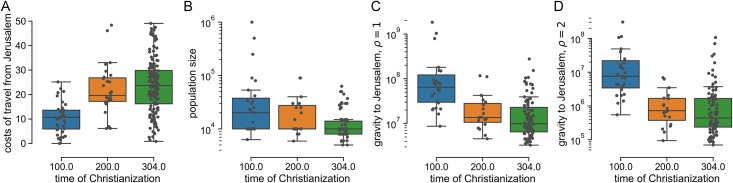
Factors of the time of Christianization. (A) Travel expense to Jerusalem, (B) population size, (C) gravity model with *ρ* = 1, and (D) gravity model with *ρ* = 2. The boxes show the median and the quartiles, the whiskers extend past the quartiles by 1.5 interquartile range.

There was a significant negative correlation between the first documented presence of Christian congregations and both the population of the city and the gravity model (*p* < 0.0001 in both cases; *N* = 135 cities; Spearman rank-order correlation; S1 Table), and significant positive relationship with the distance (i.e., cost of travel) towards Jerusalem (*p* < 0.0001 in both cases; *N* = 220 cities; Spearman rank-order correlation; S1 Table). The distributions of the four factors are summarized in Fig 2.

As can be seen in Fig 1, Christian congregations which emerged before the year 304 cover more or less the whole area of the Roman Empire, and therefore the travel cost factor from Jerusalem at this time-point in Fig 2A is equally distributed. On the other hand, all the largest cities had already been Christianized in the first century, as also shown in Fig 2B. Lastly, when we combine the population size and the cost of travel from Jerusalem in the gravity model estimation of pairwise interaction between the cities (see Methods), we get a similar statistical explanation of the date of Christianization as shown in Fig 2C and 2D. The main difference between the cost of travel from Jerusalem alone and the gravity is in the overall spatial distribution. While the map of the travel cost resembles more a traveling wavefront (see S3 Fig), the gravity to Jerusalem is large not only in the neighborhood of Jerusalem but also towards distant regional centers (see S4 Fig).

However, the evidence on the spatial distribution of Christian congregations in the first two centuries further contains clusters of smaller cities in the neighborhood of these regional centers, which seem to be seeded in advance of the traveling wavefront. These clusters of small cities have small gravity to Jerusalem, and cannot be explained by static gravity with a single point of origin. Therefore, in the next section, we will investigate the possible gravity-guided spreading process that would be able to correctly capture the gradual appearance of new sources of the spread.

### Network dynamics

The computational models of the network dynamic phenomena can quickly become sophisticated and parameter-rich, which is unsuitable in our case as the knowledge on the mechanisms of the spreading process as well as its empirical trace is not sufficient to inform the construction of such model. Therefore, we opt for the effective distance approach, which enables us to study the spreading processes on mobility networks without having to explicitly build, run and analyze the computational implementation of the model (see Methods for more details).

Normally, the effective distance is computed from a mobility network representing the flux of people between cities; however, there are no such historical data available. Therefore we approximate the mobility network by the gravity model.

**Fig 3:**
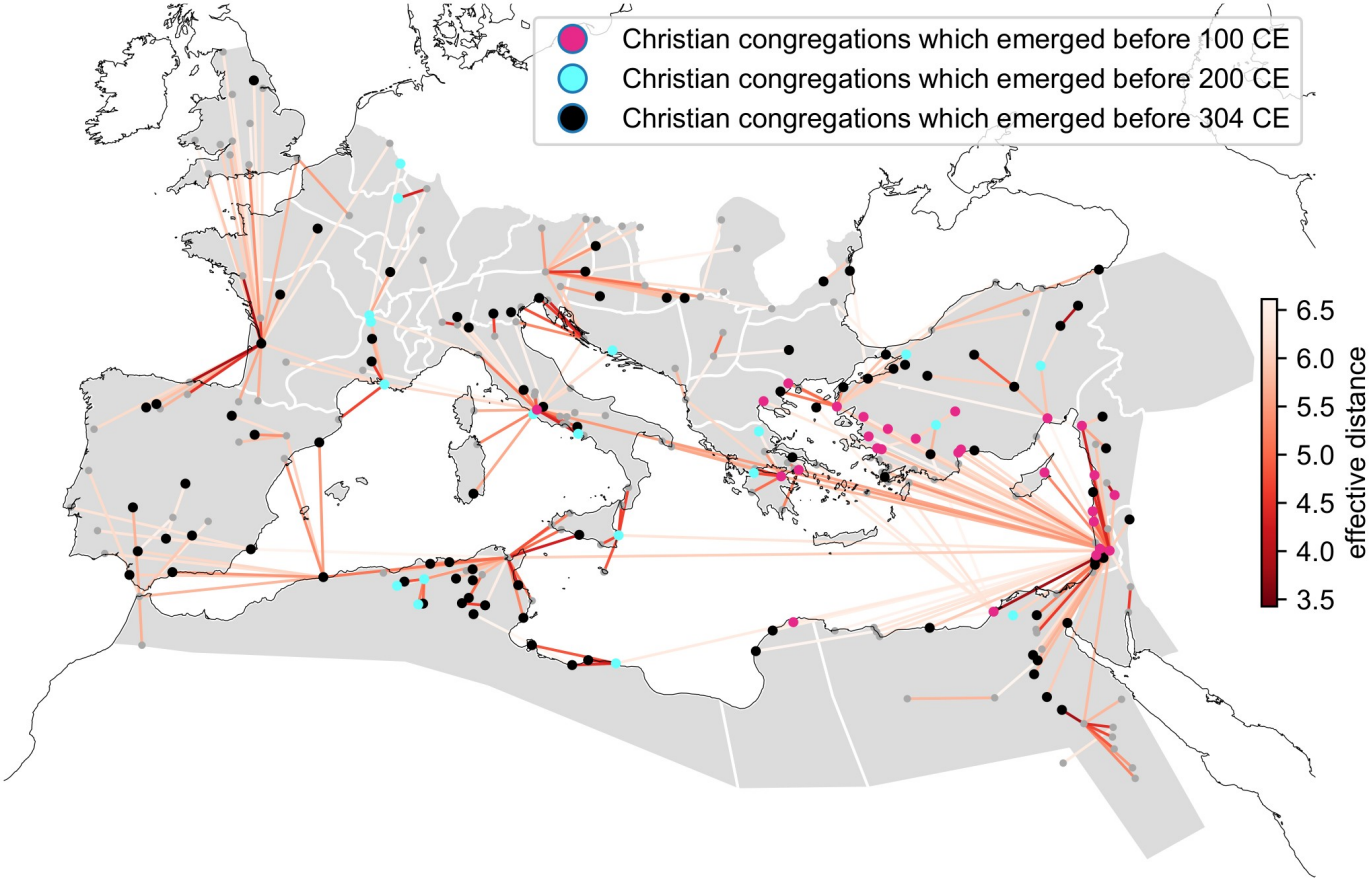
A geographical view of the shortest effective distance tree from Jerusalem.

By computing the shortest effective paths from a single point of origin, we obtain two important things: a) an expected causal tree of the spreading process and b) the effective distance to the point of origin suggesting the relative time of arrival of the spreading process. The shortest effective path tree from Jerusalem as shown in Fig 3 captures well both the direct influence of Jerusalem on the geographically close nodes, but also shows a direct connection to remote large cities such as Rome. These direct long-range connections can then facilitate the creation of remote clusters of spreading ahead of the geographical wavefront.

**Fig 4:**
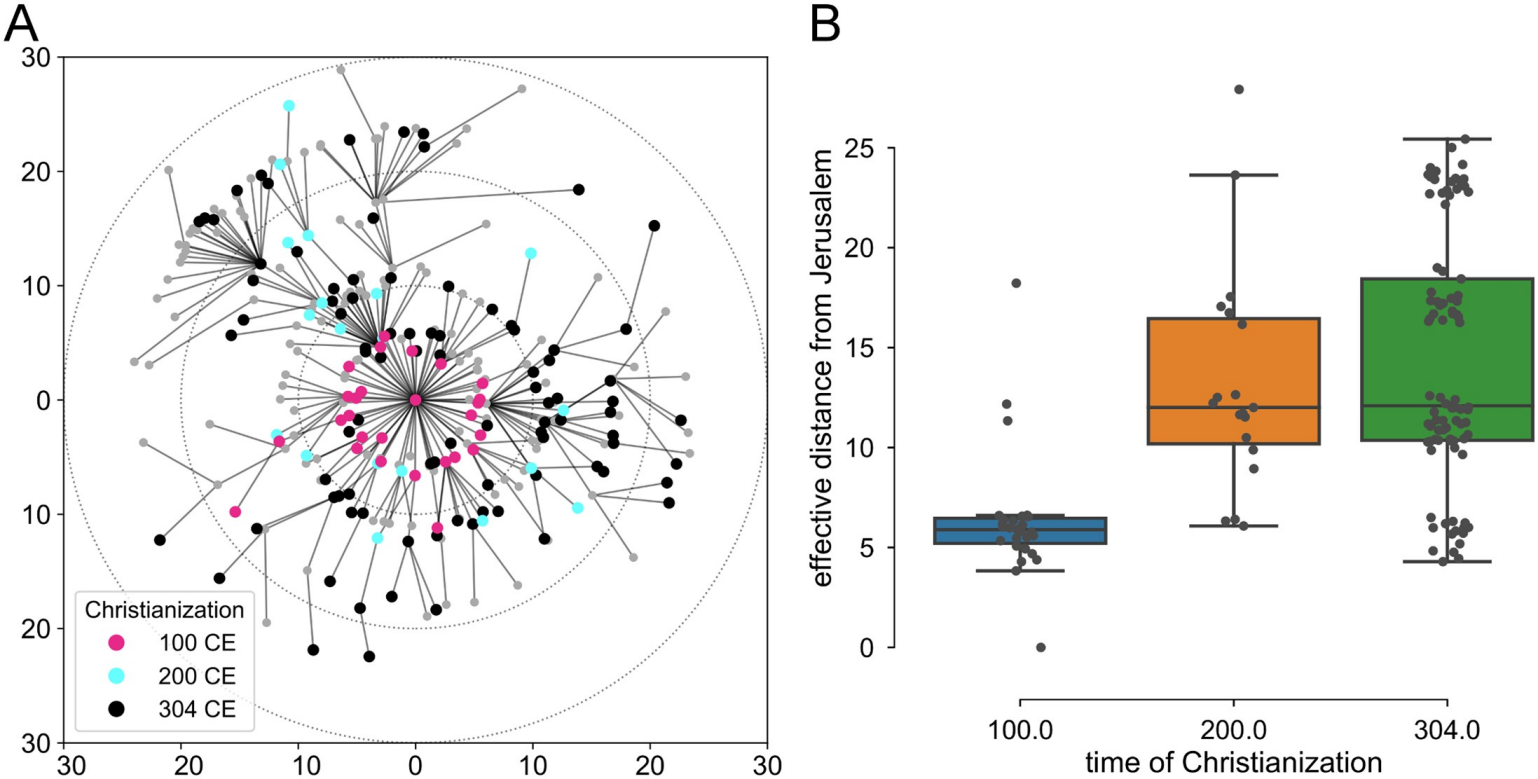
Effective distance from Jerusalem and the time of Christianization. (A) the radial distance is equal to the effective distance, colors correspond to the time of Christianization. (B) the distribution of the effective distance against the time of Christianization. The box shows the median and the quartiles, the whiskers extend past the quartiles by 1.5 interquartile range.

There was a significant positive correlation between the time of Christianization and the effective distance from Jerusalem (*r*_*s*_ = 0.43, *p* < 10^−6^; *N* = 135 cities; Spearman rank-order correlation). Again, the model captures well the first two centuries, whereas there is no significant correlation between the effective distance and the advancement of Christianization between the second and third century (Fig 4). However, on closer inspection of the shortest path tree derived from the effective distance, we found that the paths from Jerusalem are monotonic in the time of Christianization (Fig 4A). In other words, the spread of Christianity follows the temporal ordering predicted by the effective distance, but the lack of significant difference in effective distances between the second and third century suggests that the speed of advance was not uniform.

## Discussion

The cost of travel from the point of origin explains a large portion of the spatio-temporal variability in the spread of Christianity in the first two centuries (see S3 Fig). Combining the spatial constraints with the population estimates in the gravity model was shown to additionally capture Christianization of distant large cities such as Rome (see S4 Fig). Finally, the effective distance approach produced clusters in the neighborhood of these nodes, which produced an even higher fit between the model and the evidence. Our findings suggest that the nature of the spreading process was different in the third century, when the number of the documented congregations increases steeply.

We interpret the process in terms of the diffusion of innovations theory [37], which describes the adoption of an innovation over time as a S-shaped curve (sigmoid curve of a logistic function). There, the slow start of an innovation is lead by a few innovators followed by early adopters. Around the inflection point of the sigmoid curve, the innovation is adopted by an early and a late majority. Finally, there comes a saturation phase, during which the innovation slowly saturates the population of potential adopters. In our case, this framework has to be adopted with caution, as we deal with a dataset of cities and not people. However, it seems that, by the year 200, when the Christians may have represented about 0.26 percent of the total population of the empire, the spread of Christianity across Roman cities was nearing the inflection point, after which it diffused quite rapidly both geographically and in terms of numbers.

The spatial dimension of the diffusion of innovations is far less studied than other aspects of the diffusion process [38]. Considering the spatial aspect, the gravity model has been so far more successfully applied in the study of certain types of social innovations [18, 19] than in the study of technological innovations [39, 40]. Our results would suggest that the spread of the innovation was limited by the cost of travel and can be explained by the gravity model before the critical mass is reached and the adoption rate accelerates.

The combination of the gravity model and the effective distance enabled us to capture the emergence of Christian congregations in distant regional centers and their neighborhood ahead of the geographical wavefront (i.e. the expanding boundary of the area effected by spatially continuous diffusion). However, some of the cities with low effective distance to Jerusalem were Christianized much later than others with the same effective distance. This means that the spread of innovations is not automatic as is the case of disease outbreaks, where, even if an appearance of a remote cluster is possible [41–43], the disease spreads as a spatial wavefront [42]. This is true especially for the outbreaks in pre-industrial times, when the constraints on traveling were affecting the process much more directly [44, 45].

On the other hand, in some instances, the emergence of remote clusters in the model also produces some differences against the available evidence. The case of Egypt and of the neighborhood of Carthage deserves more attention in this respect, as our dataset on Christianization suggests that a considerable increase in the number of congregations occurred in these areas mainly during the third century. Contrary to this, our model, based on the effective distance, predicts many more cities in these areas to have a Christian congregation already in the first or second century. This difference between the model and the available data can mean two things: either (1) the insufficiency of the model itself (a better fit of which would require an implementation of a variable for resistance constraining the diffusion process in some areas, e.g. in the form of a cultural immunological force [46]), or (2) the existence of biases in the extant data on Christianization, somehow under-representing the evidence for these two areas. In the case of Egypt, considering how much we know about it in comparison to other parts of the Empire [47], the second possibility seems implausible.

The case of Northern Africa is different. Here we suggest that our findings might be interpreted as being in agreement with the indirect evidence that there has been a well-established spread of Christianity already by 180 CE [48]. Since our dataset on Christianization does not cover such indirect evidence, there might be a bias in it in this respect. Thus, if our model is correct, it can help to rectify this bias in the data.

Further, the rapid increase in the number of Christian congregations in the third century could be also related to some external factors that could have accelerated the spread of Christianity. Since religious and magical beliefs flourish especially under the conditions of perceived uncertainty and stress [49, 50], the rise of Christianity might have been positively stimulated by the period known as the crisis of the third century [51]. This period, marked by a political and environmental instability and several disease outbreaks [52–54], was the end of a long period of the so called Pax Romana [55], marked by Roman climate optimum [56]. This crisis could have weakened the traditional cults of the empire as ineffective [57] and could have promoted a turn to new beliefs like Christianity. Other scholars have also suggested that during this period Christianity could have benefited from prosocial behavior it promotes even toward strangers [6]. However, this hypothesis does not seem entirely persuasive [58], considering the difference between religious propositions and actual behavior [59].

Relying on a limited dataset of 31 biggest cities of the empire, Stark supported his hypothesis 3-2 (“The closer a city was to Jerusalem, the sooner a city had a Christian congregation”) by finding a significant correlation between cities having a church by 100 CE and being within 1,000 miles from Jerusalem and his hypothesis 3-4 (“Larger cities had Christian congregations sooner than smaller cities”) by finding a significant correlation between cities having a church by 100 CE and having a population of 75,000 or more [7]. With respect to both the data and the methodology, our findings represent an innovative and more robust support of a combination of these two hypotheses. We do not consider the other factors hypothesized by Stark to have an effect upon the early spatial dissemination of Christianity, such as the extent of Hellenization in a given location (hypothesis 3-3) or the presence of Jewish diaspora there (hypothesis 5-8). We have found these factors unnecessary for capturing the main properties of the process, however, this does not exclude the possibility of a future elaboration of the model to implement the additional factors too.

Stark’s usage of the geographic distance implicitly assumes a crucial role of Jerusalem in the diffusion process. This is a problematic assumption, as it seems that, especially after the destruction of the Temple, the role of Jerusalem was rather marginal [60]. The shortest effective distance tree model avoids this problem. Once the innovation arrives in a regional center, it is further driven by population size and transportation costs from this regional center to the other nodes in the network, and does not rely on the point of origin anymore. In the case of early Christianity, this is in agreement with the idea that from a very early phase cities such as Antioch or Alexandria were much more important than Jerusalem.

One of the main limitations of our study is the low reliability and temporal resolution of the data on the spread of Christianity. Since a vast majority of the data on Christianization is based on literary accounts of early Christian authors, they are necessarily tendentious [30] and, therefore, it is possible to make only very rough temporal analysis on their basis. A dataset with more precise dating would considerably improve the precision of evaluation of the model or its possible alternatives. However, as the material evidence for early Christianity is almost nonexistent, to acquire a more reliable dataset is impossible. The transportation model and the population estimates are very rough too, but considering the nature of the data on the spread of Christianity, their precision is sufficient for our purposes.

Finally, our study demonstrates that the quantitative study of spatial constraints on the diffusion of religious innovations might produce meaningful results even in areas with limited amount of primary data. With slight modifications, our model could be applied to the study of the spread of other cultural innovations in the ancient Mediterranean where we face similar problems. Moreover, with different data on transportation and population structure (such as medieval trade routes [61]), the model could be easily adapted for studying diffusion of innovations in another contexts.

## Supporting information

S1 Fig Geographical representation of the ORBIS travel model. Cities, roads, rivers and Roman provinces are shown, maritime routes ommited for brevity.
(PNG)

S2 Fig Transportation network derived from the ORBIS model. Edges represent direct cheapest connection in the ORBIS model, nodes are ORBIS sites. Only sites with population estimate available are shown, and colored by the logarithm of population size.
(PNG)

S3 Fig Travel cost to Jerusalem. Edges represent direct cheapest connection in the ORBIS model, nodes are ORBIS sites. Only sites with population estimate available are shown, and colored by the cost of travel from Jerusalem.
(PNG)

S4 Fig Gravity towards Jerusalem. Edges represent direct cheapest connection in the ORBIS model, nodes are ORBIS sites. Only sites with population estimate available are shown, and colored by the logarithm of gravity (*ρ* = 2) towards Jerusalem.
(PNG)

S5 Fig Flux fraction network after application of the 1/*M* threshold (*M* is the number of cities with population estimate). Color of the edges corresponds to the logarithm of the flux fraction between the nodes.
(PNG)

S1 File List of nodes of the travel network. Each node is identified by the ORBIS id and annotated with name of the city, geographical location, and a canonical URI within the PLEIADES project https://pleiades.stoa.org/.
(CSV)

S2 File Edge list of the travel network. Source and destination cities (nodes) are identified with unique ORBIS id. Edges are weighted by the default cost estimated by the ORBIS model.
(CSV)

S3 File Cities from ORBIS with associated population estimates. Column objectid holds an unique identifier of the city within ORBIS. The rank column contains size rank of the city from ORBIS. Column PLPATH is a canonical URI within the PLEIADES project https://pleiades.stoa.org/.
(CSV)

S4 File Geographical location and the century of emergence of the early Christian congregations. Column year contains century of first appearance of the Christianity at particular site: before year 100 (N = 54), before year 200 (N = 61), and before year 304 (N = 488).
(CSV)

S5 File Mapping of the time of emergence of early Christian congregations to the nearest ORBIS city. For every ORBIS city, only a year of the earliest congregation is retained in the year column.
(CSV)

S1 Table Spearman rank-order correlation of the static factors and the time of first documented presence of a Christian congregation. (PDF)
