# Supplementary figures and images for: Spatial constraints on the diffusion of religious innovations: The case of early Christianity in the Roman Empire
Source: PLoS One. 2018 Dec 26;13(12):e0208744. doi: 10.1371/journal.pone.0208744 (PMC6306252; doi:10.1371/journal.pone.0208744)

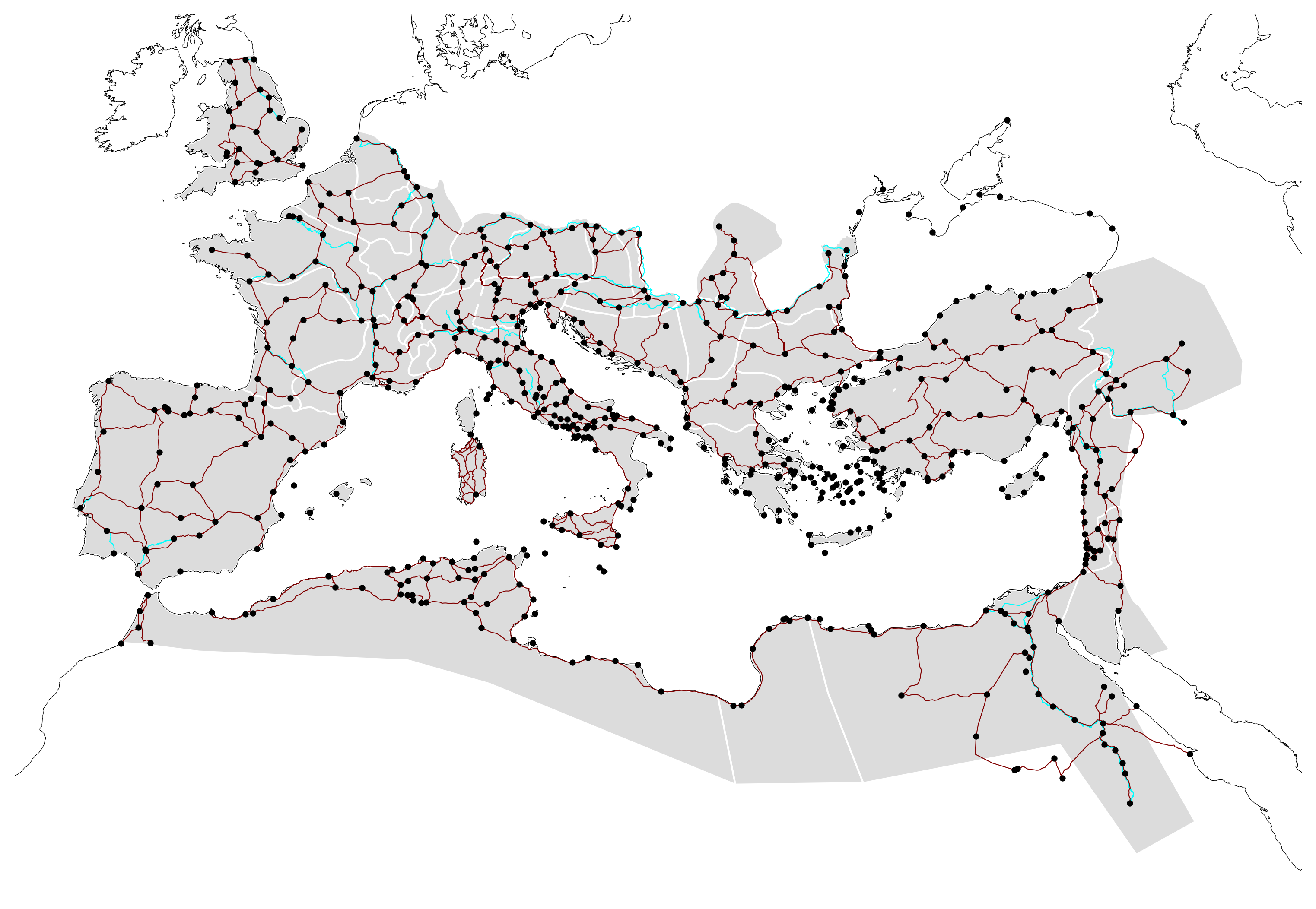

Supplement: S1 Fig — Cities, roads, rivers and Roman provinces are shown, maritime routes ommited for brevity. (PNG) [file pone.0208744.s001.png]

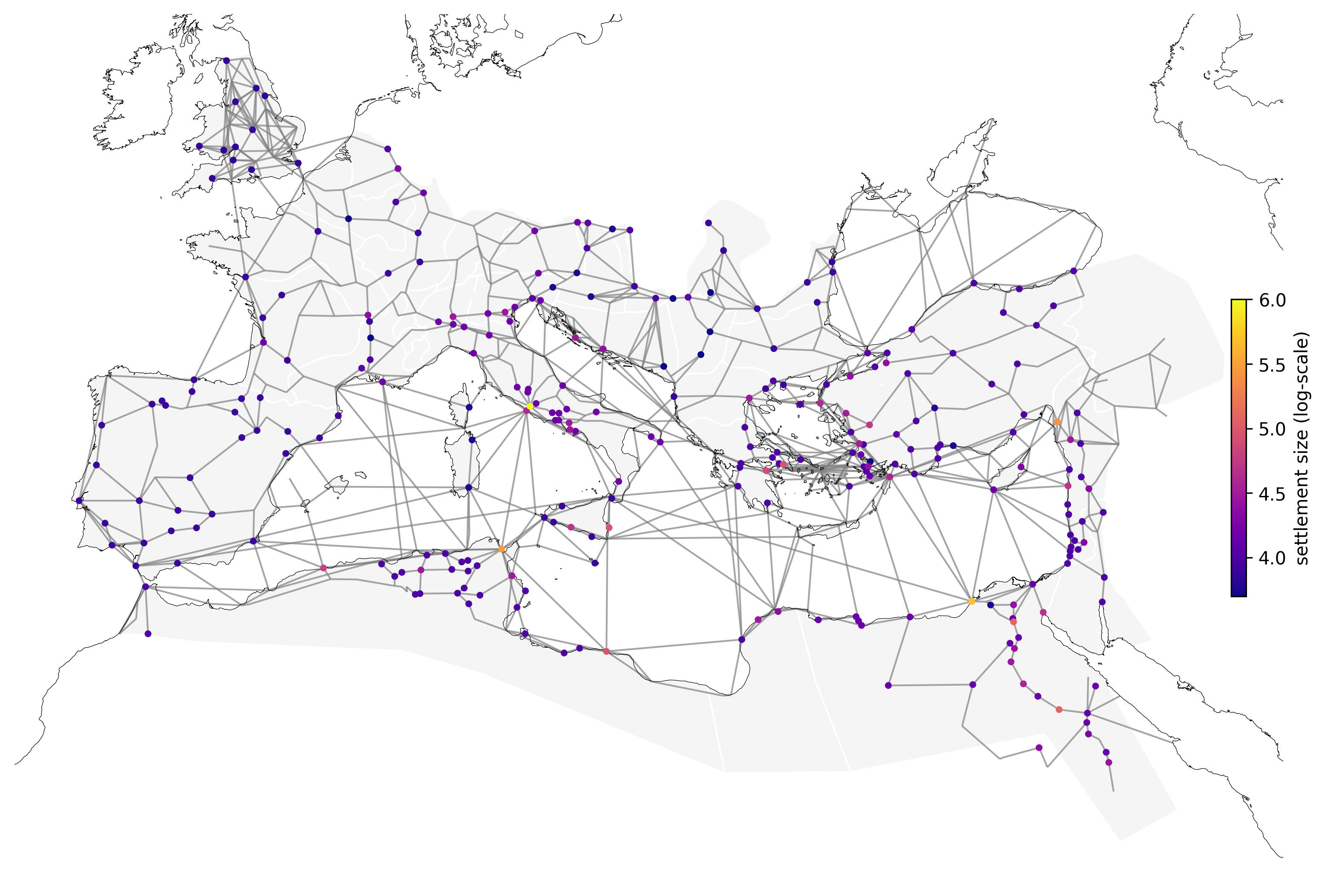

Supplement: S2 Fig — Edges represent direct cheapest connection in the ORBIS model, nodes are ORBIS sites. Only sites with population estimate available are shown, and colored by the logarithm of population size. (PNG) [file pone.0208744.s002.png]

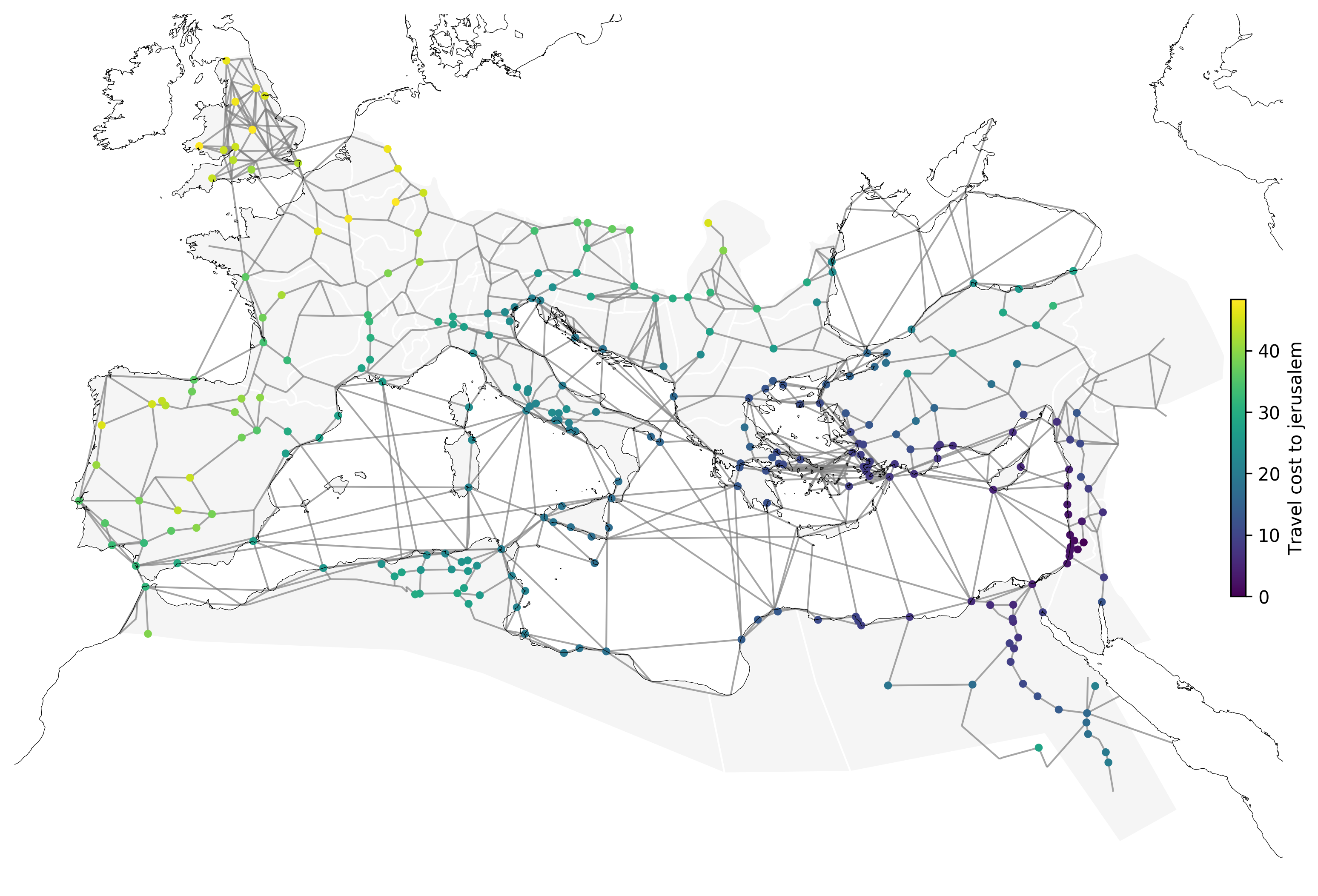

Supplement: S3 Fig — Edges represent direct cheapest connection in the ORBIS model, nodes are ORBIS sites. Only sites with population estimate available are shown, and colored by the cost of travel from Jerusalem. (PNG) [file pone.0208744.s003.png]

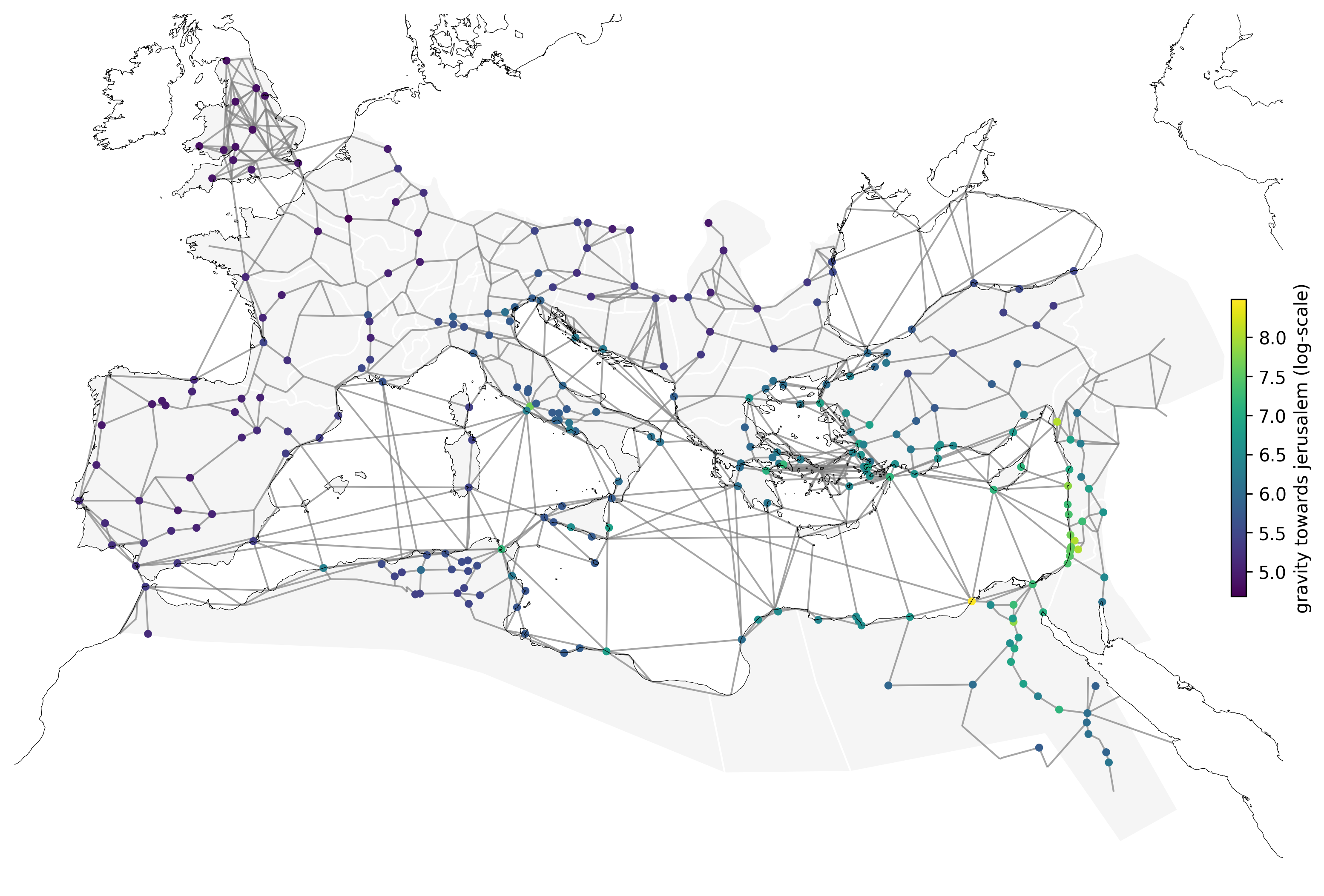

Supplement: S4 Fig — Edges represent direct cheapest connection in the ORBIS model, nodes are ORBIS sites. Only sites with population estimate available are shown, and colored by the logarithm of gravity (ρ = 2) towards Jerusalem. (PNG) [file pone.0208744.s004.png]

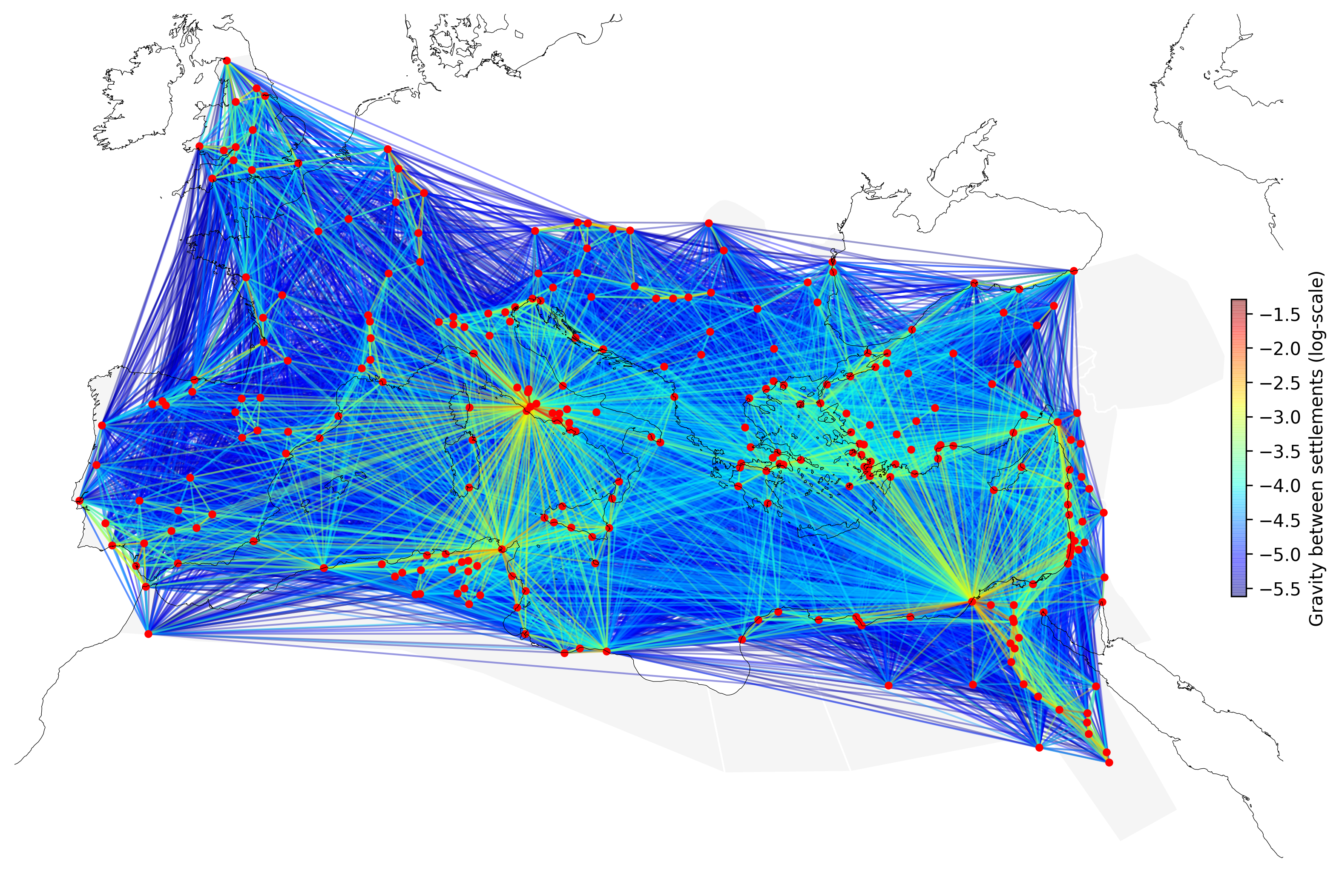

Supplement: S5 Fig — Color of the edges corresponds to the logarithm of the flux fraction between the nodes. (PNG) [file pone.0208744.s005.png]
