# Supplementary material for: Spatial constraints on the diffusion of religious innovations: The case of early Christianity in the Roman Empire
Source: PLoS One. 2018 Dec 26;13(12):e0208744. doi: 10.1371/journal.pone.0208744 (PMC6306252; doi:10.1371/journal.pone.0208744)
Supplement: S1 Table — (PDF) [file pone.0208744.s011.pdf]

Factor	r_s	P-value
distance from Jerusalem	0.42	$< 10^{-6}$
population size	-0.34	$< 10^{-4}$
gravity to Jerusalem $\rho = 1$	-0.48	$< 10^{-8}$
gravity to Jerusalem $\rho = 2$	-0.48	$< 10^{-8}$

Table 1: Spearman rank-order correlation of the static factors and the time of first documented presence of a Christian congregation.
